# Putting a Price on Nature: Ecosystem Service Value and Ecological Risk in the Dongting Lake Area, China

**DOI:** 10.3390/ijerph20054649

**Published:** 2023-03-06

**Authors:** Lisha Tang, Hualou Long, Daniel P. Aldrich

**Affiliations:** 1 School of Business, Hunan First Normal University, Changsha 410205, China; 2 Institute of Geographic Sciences and Natural Resources Research, Chinese Academy of Sciences, Beijing 100101, China; 3 School of Public Administration, Guangxi University, Nanning 530004, China; 4 Department of Political Science, School of Public Policy and Urban Affairs, Northeastern University, Boston, MA 02115, USA

**Keywords:** ecosystem service value, ecological risk evolution, spatial characteristics, correlation, Dongting Lake area

## Abstract

Understanding the relationship between ecosystem service value and ecological risk evolutions holds great theoretical and practical significance, as it helps to ensure the quality management of ecosystems and the sustainable development of human–land system interactions. We analyzed this relationship in the Dongting Lake area in China from 1995 to 2020 using data from remote sensing-interpreted land use with ArcGIS and Geoda. We used the equivalent factor method to estimate the ecosystem service value, constructed a landscape ecological risk index to quantitatively describe the ecological risk of Dongting Lake, and analyzed their correlation. The results show that: (1) over the last 25 years, the ecosystem service value decreased by 31.588 billion yuan, with higher values in the middle of the area and lower values in the surroundings—the highest value was found in forested land and the lowest was for unutilized land; (2) the ecological risk index also decreased slowly over time, from the perspective of single land use type, the ecological risk value of construction land was the lowest, followed by woodland, grassland, and cultivated land, with water area being the highest—the ecological risk level presents the distribution state of whole piece and local aggregation; and (3) the ecological risk index in Dongting Lake area demonstrated positive spatial correlation, and the spatial agglomeration of land with similar risk levels showed a decreasing trend. Areas with strong partial spatial correlations between ecosystem service value and ecological risk index are mainly distributed in the central water areas and their surrounding areas. This study investigates the rational utilization of land resources, and the sustainable development of regional ecological security in Dongting Lake area.

## 1. Introduction

Ecosystem service value and ecological risk are important types of ecological environmental assessments, which are closely related to ecological security. These assessments represent the embodiment of sustainable development of a complex human–land system [1] and can also help to ensure the successful growth of the social economy [2,3]. In recent years, there has been a continuous destruction of the ecological environment along with a gradual weakening of ecological service function [4,5] due to natural hazards caused by global climate change [6,7], accelerated population migration caused by the promotion of the integration of urban and rural development [8,9,10], and the expansion of space occupation caused by the continuous gathering of various spatial elements [11]. The sustainable development goals (SDGs) of the United Nations clearly set out to protect, restore, and promote the sustainable use of terrestrial ecosystem and sustainable forest management, to prevent and control desertification, and to stop and reverse land degradation and curb the loss of biological diversity. China has made good progress in implementing SDGs, but the ecological environment remains a challenge for China’s realization of the 2030 strategic goal of sustainable development [12,13]. Thus, preserving the ecological environment and ensuring ecological security have become common global problems and have attracted widespread attention to strengthen the construction of ecological civilization [14,15,16,17].

There has been substantial research focused on the assessment of ecosystem service value and landscape risk, including the introduction of ecosystem services into the assessment of ecological risks for development and improvement. Ecosystem services have been evaluated from different perspectives such as regional scope and time scale [18,19], and several studies have demonstrated that the forest coverage rate [20], landscape pattern [21], topographic features [22], and land use structure [23] are the main factors affecting ecosystem services. Based on the landscape ecological risk assessment (ESRISK) framework [24], some constructed an ecological security pattern [25] based on ecosystem services. In one study, ecological service degradation was considered a sign of loss, and the researchers constructed a probability representation index system from the dimensions of terrain, human behavior, ecological resilience, and landscape vulnerability [26]. The fuzzy comprehensive evaluation method [27], spatial auto-correlation analysis [28], and extensive application of the geographic information system (GIS) [29] have also been used to conduct comprehensive research on ecosystem service value and landscape ecological risk. It is also necessary to incorporate ecosystem services into an environmental risk management program and to determine ecological risks according to the ecosystem value and function [30,31].

Based on past research, we have proposed management strategies for areas with different risk levels. Overall, eco-support products and services, the value assessment of which form the base for decision-making for ecological protection, ecological regionalization, economic accounting of the ecological environment, and ecological compensation, were directly or indirectly obtained through the structure, process, and function of an ecosystem [32,33,34,35,36]. Ecological risk assessment involves evaluating the possibility of ecological effects caused by exposure to multiple risk stressors of the ecosystem and its components, which then forms the premise for risk control [37,38,39,40]. Although numerous studies have estimated the ecological service value and its distribution in time and space, research focusing on the effective correlation between the change of the ecological environment and the sustainable development of the system is relatively sparse. Effective analysis would need to consider the evolution of ecological service value and risks to propose management strategies for different risk levels through assessing their correlations. 

Toward this end, in the present study, we used Landsat Thematic Mapper (TM) remote sensing images of the Dongting Lake area in China from 1995 to 2020 as the basic data source to obtain the land cover data of the region through human–computer interactive interpretation. The equivalent factor method and the spatial analysis model were then used to analyze the spatial-temporal distribution and changes of ecosystem services in the Dongting Lake area. As the title of our paper indicates, we have used a landscape ecological risk assessment model to investigate the spatial-temporal variation of the ecological risk index. Our analysis of the correlation between the ecosystem service value and the ecological risk assessment puts a “price” on nature, providing a framework for more efficient utilization of land resources. Doing so will help establish more stable regional ecological security patterns in the Dongting Lake area and propel the sustainable development of regional ecology.

## 2. Materials and Methods

### 2.1. Research Area

**Figure 1:**
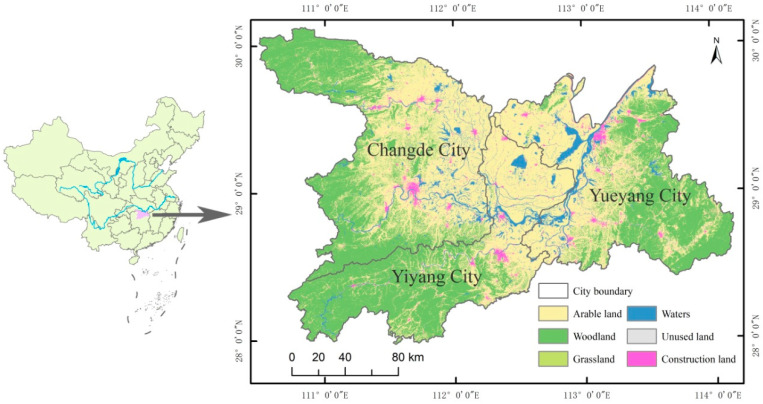
Location of the study area and land use map in 2020.

Dongting Lake, the second largest freshwater lake in China, is in the Northern Hunan Province (28°00′–30°20′ N, 110°30′–114°30′ E), south of Jingjiang River in the reach of the Changjiang River (Figure 1). Thus, Dongting Lake is an important regulating lake and ecological functional area in the middle and lower reaches of the Changjiang River. The protection of its ecological environment plays an important role in ensuring the safety of water resources in the Changjiang river basin and maintaining the ecological balance of the Dongting Lake area. The area of Dongting Lake selected for this study is mainly from the Changde, Yiyang, and Yueyang of Hunan Province, with a total area of 453.7 km^2^. The study area has a subtropical monsoon humid climate with typical continental climate characteristics. In this area, there are developed water systems, four distinct seasons, and abundant rainfall. The annual precipitation is 1300–1600 mm, the average annual temperature is 16–20 °C, and the altitude is 30–50 m. The land resources are mainly mountains and hills, the topography is very undulating, the forest coverage rate is high, and rice is the main crop grown in the region.

### 2.2. Data Source and Preprocessing

Six Landsat TM/Enhanced Thematic Mapper (ETM) remote sensing images of land use in the study area from 1995 to 2020 were derived from the Data Center for Resources and Environmental Sciences, Chinese Academy of Sciences. We chose Landsat TM images of 1995, 2000 and 2005, Landsat EMT+ images of 2010 and Landsat OLI remote sensing images of 2015 and 2020 as remote sensing images, the distinguishability of which is 30 m × 30 m. Social and economic data were obtained from the *Chinese Statistical Yearbook, Hunan Provincial Statistical Yearbook*, *Hunan Provincial Prefecture-level Statistical Yearbook,* and *Compilation of Cost and Income Data of National Agricultural Products*; other relevant data were calculated by basic statistics.

After integrating the scale effects of the study area and the minimum modifiable unit on the measured results and several tests, we divided the landscape pattern of Dongting Lake into grids of 5 km × 5 km by using Create fishnet, resulting in 2019 evaluation grids. Based on these grids, the ecosystem service value of the study area was calculated, and the ecological risk value was calculated in Fragstats 4.2 software. The value was assigned to the center point of the evaluation cell to analyze the spatial distribution characteristics.

### 2.3. Ecosystem Service Value Calculations

Based on the value equivalent factor per unit area [41], we adjusted the equivalent factor table of China’s ecosystem service value by combining the revised table [42] with the actual situation and related achievements [43,44] of the study area. Ecosystem services were divided into four primary categories: supply service, regulation service, support service, and cultural service. Each of these categories was further divided into 11 subcategories: food production, raw material production, water supply, gas regulation, climate regulation, environmental purification, hydrological regulation, soil conservation, nutrient cycle maintenance, biodiversity, and esthetic landscape.

#### 2.3.1. Equivalent Coefficient Correction

Due to the different characteristics of environment, climate, and economy in different study areas, the value of ecosystem services varies greatly and is constantly changing. Therefore, to improve the accuracy of the calculation results, in this study, we further analyzed and determined the dynamic factors of grain coefficient, social coefficient, and regional difference coefficient, and carried out the spatio-temporal dynamic correction of the ecosystem service value in the Dongting Lake area. The formulae used for correction are as follows:

(1) $$P_{t}=C_{t^{\prime }}/C_{t}$$

(2) $$D_{t}=l_{t^{\prime }}/l_{t}$$

(3) $$l=l_{1}M_{1}+l_{2}M_{2}$$

(4) $$Q_{t}=NNP_{t^{\prime }}/NNP_{t}$$

(5) $$NNP=3000\left[1-e^{-0.000969\left(R-20\right)}\right]$$

$P_{t}$ represents the corrected grain coefficient in year *t*,$\;C_{t^{\prime }}$ represents the average grain output of the Dongting Lake area (kg/hm^2^), and $\;C_{t}$ represents the national average grain output (kg/hm^2^). $D_{t}$ represents the corrected coefficient of social development in the year *t*, $l_{t^{\prime }}$ represents the social development coefficient of the Dongting Lake area, and $l_{t}$ represents the social development coefficient of the whole country. l represents the social development coefficient related to the actual willingness to pay, *l*_1_ is the coefficient of urban social development, *M*_1_ represents the proportion of the urban population, *l*_2_ is the rural social development coefficient, and *M*_2_ represents the proportion of the rural population. $Q_{t}$ represents the corrected coefficient of regional difference in the year *t*, $NNP_{t^{\prime }}$ represents the net primary production potential of natural vegetation(t·hm^−2^·a^−1^), and $NNP_{t}$ represents the average net primary productivity of all types of vegetation (t·hm^−2^·a^−1^). In the formula to calculate *NNP*, *R* represents the actual evapotranspiration (mm) in the study area within a year.

#### 2.3.2. Evaluation of Ecosystem Service Value

Based on the ecosystem service value proposed above, 1/7 of the economic value of 1 hm^2^ farmland grain production was taken as the value of ecosystem services for a standard equivalent factor [45]. Paddy fields are the main cultivated land in the study area, and rice output accounts for more than 90% of the output among the three major cultivated grains. Therefore, the economic value of rice was used to replace the economic value of farmland grain production. In 2020, the rice output per unit area in Dongting Lake was 6608 kg/hm^2^, and the average price of rice was 2.59 yuan/kg. Therefore, the equivalent factor of an ecosystem service value in the study area was set to 2445.21 yuan/kg. The calculation formula used for the ecosystem service value in the Dongting Lake district is as follows:

(6) $$ESV=\sum \left(A_{k}\times VC_{k}\times P_{t}\times D_{t}\times Q_{t}\right)$$

(7) $$ESV_{f}=\sum \left(A_{k}\times VC_{fk}\times P_{t}\times D_{t}\times Q_{t}\right)$$

(8) $$VC_{k}=\sum VC_{fk}$$

where *ESV* is the total ecosystem service value of the study area (yuan), *A_k_* is the area of land use type *k* (hm^2^), and *VC_k_* is the ecosystem service value coefficient per unit of class *k* land (yuan/hm^2^). *P_t_*, *D_t_*, and *Q_t_* represent the corrected grain coefficient, social development coefficient, and regional difference coefficient, respectively, in the year *t*. *ESV_f_* is the value of service function of item *f* in the ecosystem (yuan), *VC_fk_* is the service function value coefficient of item *f* for land use type k (yuan/hm^2^).

### 2.4. Assessment of Landscape Ecological Risk

Starting from the landscape pattern, we used the grid method to divide the risk units and constructed the landscape ecological risk index for spatial interpolation so as to quantitatively describe and evaluate the ecological risk level of the community. The formulae used for this calculation are as follows:

(9) $$ERI_{k}=\sum_{i=1}^{n}\frac{A_{ki}}{A_{k}}\times R_{i}$$

(10) $$R_{i}=E_{i}\times F_{i}$$

(11) $$E_{i}=aS_{i}\times bN_{i}\times cT_{i}$$

where $\;ERI_{k}$ is the landscape ecological risk index of quadrat *k*, $A_{ki}$ is the landscape area of item *i* within the quadrat, $A_{k}$ is the interior landscape area of the item quadrat, $S_{i}$ is the fragmentation index of the landscape item *i*, $N_{i}$ is the separation index, $T_{i}$ is the dominance index, $E_{i}$ is the disturbance index, $F_{i}$ is the vulnerability index, and $R_{i}$ is the loss index (Table 1). In addition, *a*, *b*, and *c* are the index weights for calculating the landscape interference degree index *E_i_*, which were assigned values of 0.5, 0.3, and 0.2, respectively. Landscape vulnerability refers to the ability of landscape types to resist external disturbances. Combined with the actual situation of the study area, unutilized land, water area, cultivated land, grassland, woodland, and construction land were assigned values of 6, 5, 4, 3, 2, and 1, respectively. After normalization, the vulnerability index *F_i_* of each landscape type was 0.2857, 0.2381, 0.1905, 0.1429, 0.0952, and 0.0476, respectively.

### 2.5. Spatial Autocorrelation Model

Spatial autocorrelation refers to the degree of correlation between an attribute of an element in each geographic spatial area and the same attribute in adjacent spatial areas, including overall spatial autocorrelation and partial spatial auto correlation [46]. In this study, we used the overall spatial autocorrelation coefficient Moran’s I to reflect the overall spatial correlation. The bivariate local spatial autocorrelation model was used to explore the spatial relationship between the ecosystem service value and ecological risk index in the Dongting Lake area. The specific formula is as follows:

(12) $$GI=\frac{\sum_{i=1}^{n}\sum_{j=1}^{n}W_{ij}\left(x_{i}-\bar{x}\right)\left(x_{j}-\bar{x}\right)}{\sum_{i=1}^{n}\sum_{j=1}^{n}W_{ij}{\left(x_{i}-\bar{x}\right)}^{2}}$$

(13) $$LI=Z_{i}\sum W_{ij}Z_{j}$$

where *GI* is the overall spatial auto correlation, *x_i_* and *x_j_* are the ERI of unit *i* and unit *j* in the grid, respectively, *n* is the total number of grids in the study area, *W_ij_* is the matrix of space weight, and $\bar{x}$ is the average value of *ERI*. *LI* is the partial spatial autocorrelation, $Z_{i}$ and $Z_{j}$ are the normalized values of spatial units *i* and *j*, respectively, and $W_{ij}$ is the space weight.

## 3. Results

### 3.1. Spatial and Temporal Distribution of the Ecosystem Service Value in the Dongting Lake Area

#### 3.1.1. Analysis of Temporal Changes in Ecosystem Service Value

Using 5 years as the time scale and based on an analysis carried out by ArcGIS software, this study combined land use data and the ecosystem service value scale to estimate the regional ecosystem service value of Dongting Lake from 1995 to 2020 (Table 2). In the past 25 years, the ecosystem service value of the Dongting Lake area showed a pattern of negative growth, decreasing by 31.588 billion yuan, with a growth rate of −11.68%. From the perspective of different land use types, the ecosystem service value of cultivated land, forest land, grassland, and water area decreased continuously with change rates of −8.06%, −8.63%, −18.46%, and −16.14%, respectively. The service value of unutilized land fluctuated, although this change was not particularly obvious. The land use in the Dongting Lake area is mainly cultivated land and forest land, each accounting for more than 40% of the total land, followed by water area. In recent years, due to the acceleration of urbanization, the area of forest land, grassland, water area, and cultivated land has been continuously declining, along with their corresponding ecosystem service values. Therefore, the total value of ecosystem services in the Dongting Lake area has decreased over time. The ecosystem service value of each land use type in the study area was in the order of forest land > water area > cultivated land > grassland > unused land.

In the Dongting Lake ecosystem from 1995 to 2020, among all service types, the largest contribution rate was adjustment service, followed by support service, supply service, and cultural service (Table 3), and the value of each type of service showed a continuous decrease overall. From the perspective of the secondary classification of ecosystem service functions, the value of each function, from high to low, was in the following order: hydrological regulation, climate regulation, gas regulation, soil conservation, biodiversity, environmental purification, food production, esthetic landscape, raw material production, maintenance of nutrient cycle, and water supply. More than 40% of the Dongting Lake area is forest land, and the ecosystem service function of forest land is mainly the regulation service. With the decrease of forest land area, the values of hydrological regulation, climate regulation, gas regulation, and environmental purification services in the study area have been continuously decreasing. Food production and water supply are mainly affected by the change of cultivated land area. The Dongting Lake area is rich in water resources and the main cultivated crop is rice. The continuous reduction of paddy area and water demand has led to a reduction in the value of the food production service by 862 million yuan. The water supply gap from 1995 to 2015 has been decreasing continuously; however, in 2020, the water supply gap increased due to the increase of cultivated land area. Therefore, the raw material production, soil conservation, nutrient cycle maintenance, biodiversity, and esthetic landscape ecosystem service values decreased.

#### 3.1.2. Spatial Change of Ecosystem Service Value

**Figure 2:**
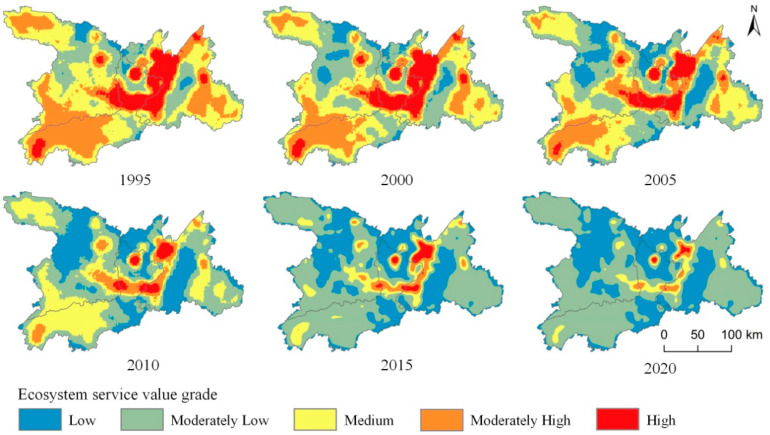
Spatial pattern of ecosystem service value per unit area in the Dongting Lake area from 1995 to 2020.

This study used the grid method to calculate and evaluate the ecosystem service value per unit area of the community, and then obtained the distribution of ecosystem service value in Dongting Lake by center point assignment and spatial interpolation. Finally, it was divided into five levels (Figure 2) according to the geometric interval classification and combined with the actual situation of the study area: low (0, 1], moderately low (1, 2], medium (2, 3], moderately high (3, 4], and high (4, ∞). The ecosystem service value of the Dongting Lake area was mainly identified as high, moderately high, and medium from 1995 to 2000. Since 2005, the decline of woodland, grassland, and cultivated land areas in the Dongting Lake area has been accelerating while the construction land area has been increasing. Therefore, in 2005, the proportion of high or moderately high ecosystem service value areas in the Dongting Lake area has been decreasing, and the major ecosystem service value was moderately high or medium. During 2010–2020, as the urbanization process accelerated, the value of ecosystem services in the study area had been continuously reducing, mainly at a medium or moderately low level. The central area of Dongting Lake has a dense water network and abundant resources, especially in the west of Yueyang County, Xiangyin County, Yuanjiang City, and Hanshou County. In the past 25 years, the ecosystem service value in this area has remained high and moderately high, with relatively the least amount of change in the total value.

### 3.2. Spatial-Temporal Distribution Characteristics of Landscape Ecological Risk

#### 3.2.1. Temporal Variation Characteristics of Landscape Ecological Risk

**Figure 3:**
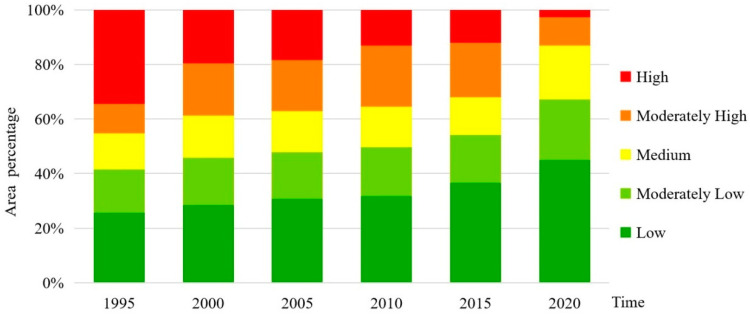
Change trend of ecological risk levels in the Dongting Lake area from 1995 to 2020.

We used ArcGIS 10.8 to construct a landscape risk index so as to obtain the ecological risk index values of Dongting Lake in 1995, 2000, 2005, 2010, 2015, and 2020. The mean values of the whole area were 0.0408, 0.0376, 0.0367, 0.0355, 0.0340, and 0.0290 for these years, respectively, showing a slow decreasing trend. From the perspective of single land use types, construction land had the smallest landscape risk value, followed by woodland, grassland, and cultivated land, whereas water area had the largest landscape risk value. As shown in Figure 3, during 1995–2020, the transformation of ecological risk levels in the study area went from high and moderately high risk to medium, moderately low, and low risk. Among these areas, the areas with high ecological risk decreased the most, by 31.54% in 25 years, and the areas with low ecological risk increased the most, by 19.37%. Areas with other risk levels did not change substantially during this time.

#### 3.2.2. Spatial Change Characteristics of Landscape Ecological Risk

**Figure 4:**
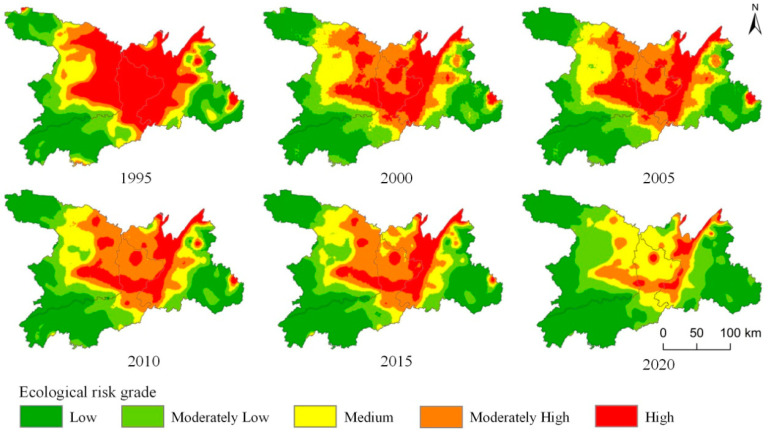
Spatial pattern of ecological risk distribution in Dongting Lake area from 1995 to 2020.

In ArcGIS 10.8 software, we used the Kriging method of spatial interpolation to analyze the ecological risk index of grid units in the research area by spatial interpolation. The landscape risk was divided into five levels: low ecological risk (0, 0.026), moderately low ecological risk (0.026, 0.034), medium ecological risk (0.034, 0.042), high ecological risk (0.042, 0.050), and moderately high ecological risk (0.050, ∞). As shown in Figure 4, the ecological risk levels in the Dongting Lake area from 1995 to 2020 presented a state of general integration with minimal differences. Over the 25 years, the overall ecological risk in the Dongting Lake area was higher in the middle and lower in the east and west, and the change rate of ecological risk was in the order of high risk > low risk > moderately low risk > medium risk > moderately high risk. Areas with high ecological risk decreased year by year, while the areas with moderately high ecological risk increased first and then decreased, although the magnitude of change was small. The areas with high and moderately high ecological risk were mainly distributed in the east of Changde City, the northeast of Yiyang City, and the west of Yueyang City, where water area and cultivated land are the main land types. The water areas are vulnerable to risks; therefore, it is necessary to strengthen ecological protection and monitoring on water areas to reduce the impact of human activities. Areas with low and moderately low ecological risk are increasing year by year, mainly distributed in the west of Changde City, the southwest of Yiyang City, and the east of Yueyang City. These areas accounted for an increase of 19.37% and 6.54% from 25.52% and 15.72% in 1995, to 44.90% and 22.26% in 2020, respectively. Land use types in low and moderately low ecological risk areas were mainly woodland and grassland, which have been largely converted into construction land. With the steady increase and orderly development of urban construction land and the implementation of relevant ecological environmental protection policies, the ecological risk index has been decreasing. The spatial change of middle ecological risk areas was not evident.

### 3.3. Correlation Analysis between Ecosystem Services and Ecological Risks

#### 3.3.1. Overall Spatial Autocorrelation

**Figure 5:**
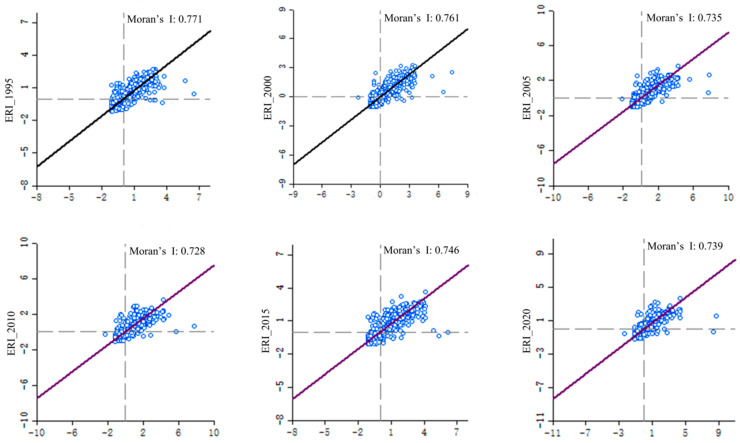
Moran scatter of ecological risk index of Dongting Lake area.

Based on ArcGIS 10.8, we calculated the Moran’s I value of the ecological risk index of the 2019 grids in the Dongting Lake area in 1995, 2000, 2005, 2010, 2015, and 2020. From 1995 to 2020, the Moran’s I values of these six phases in the study area were 0.771, 0.761, 0.735, 0.728, 0.746, and 0.739, respectively (Figure 5). The values of Moran’s I were all greater than 0 with a *p* value of 0.001. Monte Carlo simulation demonstrated that the spatial distribution of the ecological risk index in the Dongting Lake area shows a positive correlation. From 2000 to 2015, the values of Moran’s I showed an overall downward trend, indicating that the degree of autocorrelation was relatively weakened. In addition, the spatial agglomeration of sample plots, which are of similar land use ecological risk levels, showed an overall decreasing trend.

#### 3.3.2. Local Spatial Autocorrelation

**Figure 6:**
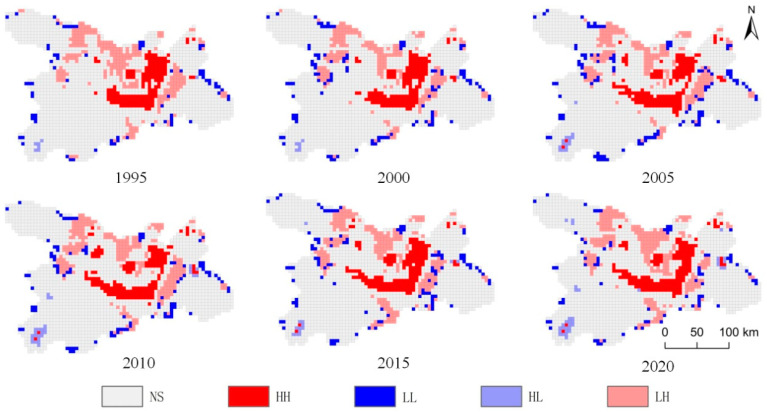
Spatial autocorrelation between ecosystem service value per unit area and ecological risk index from 1995 to 2020: NS, not significant; HH, high value/high risk; LL, low value/low risk; HL, high value/low risk; LH, low value/high risk.

Based on the GeoDa bivariate spatial autocorrelation model, we analyzed the spatial correlation between the ecosystem service value and landscape ecological risk index in the Dongting Lake area. As shown in Figure 6, the index could be divided into high value/high risk, high value/low risk, low value/high risk, low value/low risk, and insignificant. The results showed that areas with significant correlations were mainly those in the middle, including the west of Changde City, the southwest of Yiyang City, and the east of Yueyang City. The water area of Dongting Lake showed a high value/high risk distribution, while the surroundings were classified as low value/high risk areas.

High value/high risk areas were mainly distributed in Western Yueyang County, Xiangyin County, Yuanjiang City, and Hanshou County. Water areas can provide high ecosystem service value. Turning lakes into fields accelerated the process of paludification, which resulted in a series of events: the continuous reduction of water area, the decline of ecosystem service value, the decline of ecological environment quality, frequent flood disasters, biodiversity decline, and great damage to aquatic resources. After the catastrophic flood in 1998, Hunan Province began to implement the “4350” project, and returned the farmland to lake, which alleviated the declining rate of the Dongting Lake area, reduced the declining rate of the ecosystem service value of the low value/high risk state, and reduced the incidence of flood disasters. The declining rate of the low value/high risk ecosystem service reduced over time, along with the incidence of flood disasters.

Low value/low risk areas were scattered, small, and mainly represented by construction land, greatly affected by human activities. The impact of human activities on the regional ecological environment resulted in a stable and continuously low value of ecosystem services, leading to a low ecological risk.

High value/low risk areas were mainly distributed in the west of Anhua County and the east of Yueyang County. This region is rich in forest land resources and thus had the highest ecosystem service value compared with that of other land use types. Moreover, these areas are less affected by climate and showed little change in the ecosystem service value. These areas were generally transformed to grassland within a short time and thus are not prone to ecological risks.

Low value/high risk areas were mainly distributed in the surroundings of high value/high risk areas. These areas are greatly affected by the agglomeration effect of land with high ecosystem service value, mainly constituting cultivated land with lower ecosystem service value compared with those of water area and forest land. In recent years, due to the accelerated process of urbanization, construction land has crowded into cultivated land, resulting in the continuous reduction of cultivated land area, which is mainly transformed into construction land or waste land with a high ecological risk.

Finally, insignificant areas are those in which the agglomeration relationship between the ecosystem service value per unit and landscape ecological risk intensity coefficient was not clear. This type of area accounted for approximately 65% of the total study area, mainly represented by woodland and cultivated land, and the distribution of ecosystem service value and ecological risk index was relatively balanced.

## 4. Conclusions and Discussion

### 4.1. Conclusions

Based on the land use data of the Dongting Lake area from 1995 to 2020, this study evaluated the ecosystem service value and ecological risk index, analyzed the spatio-temporal evolution characteristics in the area, and further explored the relationship between the two factors using a spatial regression model.

Overall, the temporal and spatial evolution of ecosystem service value in the Dongting Lake area showed significant changes. From the perspective of time evolution, the total ecosystem service value of the Dongting Lake area showed a decreasing trend from 1995 to 2020. The ecosystem service value of cultivated land, forest land, grassland, and water area has been decreasing continuously, and the ecosystem service value of unutilized land fluctuated. From the perspective of spatial evolution, the ecosystem service value of the Dongting Lake area was mainly categorized as high, moderately high, and medium from 1995 to 2000, whereas that of 2004 was categorized as moderately high and medium. As the urbanization process accelerated from 2010 to 2020, the ecosystem service value of the Dongting Lake area decreased continuously, mainly represented by medium and moderately low values.

The ecological risk index of the Dongting Lake area showed a slowly decreasing trend. In terms of time, from 1995 to 2020, construction land exhibited the smallest landscape risk value from the perspective of single land use type, while the water area and cultivated land had the largest risk values. The transformation of ecological risk levels presented a change pattern from high and moderately high risk to medium, moderately low, and low risk. The area of high ecological risk declined the most, while the area of low ecological risk showed the greatest increase. This area presented a state of overall integration with little difference in space, specifically, the risk was high in the northwest and low in the southeast. The ecological risk levels changed in the following order: high risk > low risk > moderately low risk > medium risk > moderately high risk.

The correlation between ecosystem services and ecological risks in the Dongting Lake area mainly include overall autocorrelation and partial spatial autocorrelation patterns. The overall autocorrelation of the ecological risk index showed that in the last 25 years, the Moran’s I value has been continuously decreasing, especially after the implementation of “4350” project in Hunan Province. The project seeks to return farmland to the lake, build polder land and resettle people in residential towns, and constantly restore the water area of Dongting Lake area.It makes the spatial agglomeration of sample plots with similar levels of ecological risk of land use show an overall decreasing trend. Through bivariate spatial autocorrelation, this study analyzed the spatial correlation between the ecosystem service value and landscape ecological risk index in the Dongting Lake area. The areas showing significant correlations were mainly distributed in the middle of the region, namely the west of Changde City, southwest of Yiyang City, and east of Yueyang City. The water area of Dongting Lake showed an obvious high value/high risk distribution, whereas most of the surrounding areas showed a low value/high risk distribution.

### 4.2. Discussion

From 1995 to 2020, the ecological risk index of the Dongting Lake area has been decreasing continuously, whereas the value of ecosystem services showed a declining trend. Urbanization, human activities, climate change, public policies and other impacts were responsible for the change of the ecological environment to a certain extent, and these effects further aggravated the existing challenges in this area. The high and moderately high ecosystem service value areas and the low and moderately low risk areas of Dongting Lake are relatively less affected by human activities and disturbances, but it will be difficult to restore these areas once they have been damaged. Therefore, the industrial layout should be reasonably planned according to ecological suitability. Moreover, focus should be placed on the development of agriculture, forestry, animal husbandry, and fishing, based on the primary industries, to protect the ecological environment while improving economic benefits. In the middle ecosystem service value areas and the middle risk areas of Dongting Lake area, the status quo should be maintained as far as possible to reduce the future ecological risk. Industrial transformation and upgrading should be accelerated according to local conditions to reduce and ban the pollution and resource intensive industries. The farmland should be returned to forest or grassland to promote sustainable development. Affected by natural conditions and human activities, the low and moderately low ecosystem service value areas and the high and moderately high risk areas in Dongting Lake exhibit the characteristics of low value, high ecological risk, and susceptibility to changes in the ecological environment. Therefore, it is necessary to strengthen the protection of the original landscape, establish an early warning mechanism for ecological risks, formulate standards for ecological restoration and reconstruction, and strengthen ecological security barriers. In addition, relevant government departments should continue to adhere to the strategy of regional ecological environmental protection and sustainable social and economic development, adjust the relationship between resources and the ecological environment, strengthen the protection of ecological conservation areas, implement the delineation and implementation of regional ecological conservation red lines, and strengthen the control over land and space use.

## Figures and Tables

**Table 1:** Method for constructing the landscape pattern index.

Index	Formula	Ecological Meaning
Landscape fragment, $S_{i}$	$S_{i}=\frac{n_{i}}{A_{i}}$	$S_{i}$ represents the fragmentation degree of landscape segmentation; the greater the value, the lower the stability of the corresponding landscape ecosystem. $n_{i}$ represents the number of patches of landscape type *i* and $A_{i}$ represents the total area of landscape type *I*; the greater the value of $S_{i}$, the greater the degree of fragmentation.
Landscape separation	$N_{i}=\frac{A}{2A_{i}}\sqrt{\frac{n_{i}}{A}}$	$N_{i}$ represents the degree of separation of patch distribution in the same landscape type; the greater the value, the more complex the corresponding landscape spatial distribution and the higher the degree of fragmentation. $n_{i}$ represents the number of patches of landscape type *i*, A represents the total area of the landscape, and $A_{i}$ represents the total area of landscape type *i.*
Landscape dominance, $T_{i}$	$T_{i}=\left(M_{i}\times W_{i}\times R_{i}\right)/3$	$M_{i}$ is the number of grids appearing in patch *i*/the total number of grids, $W_{i}$ is the number of patches *i*/the total number of patches, and $R_{i}$ is the area of patch *i*/total quadrat area.
Landscape interference, $E_{i}$	Delphi and Normalization	$E_{i}$ represents the influence of human interference on the region; the smaller the value, the more beneficial to the survival of organisms.
Landscape fragility, $F_{i}$	$E_{i}=aS_{i}\times bN_{i}\times cT_{i}$	$F_{i}$ represents the sensitivity of different landscape types to external disturbances; the higher the value, the higher the ecological risk.
Landscape loss degree, $R_{i}$	$R_{i}=E_{i}\times F_{i}$	$\;R_{i}$ represents the difference of ecological loss suffered by different types of landscapes upon interference, namely the degree of loss of natural attributes.

**Table 2:** Structure and corresponding changes of ecosystem service value (ESV) in the Dongting Lake area from 1995 to 2020.

Landscape Type/ Value Division	ESV/100 Million						Change of ESV/100 Million	Rate of Change (%)
	1995	2000	2005	2010	2015	2020		
Cultivated land	237.68	233.35	229.10	223.65	221.15	218.54	−19.15	−8.06
Forest land	1351.43	1326.80	1303.88	1273.61	1255.04	1234.76	−116.67	−8.63
Grassland	0.40	0.36	0.26	0.32	0.36	0.33	−0.07	−18.46
Water area	1114.95	1093.92	1075.75	1045.36	1044.47	934.97	−179.99	−16.14
Unutilized land	0.04	0.04	0.05	0.05	0.04	0.04	0.00	−1.70
Construction land	0.00	0.00	0.00	0.00	0.00	0.00	0.00	0.00
Total value	2704.51	2654.47	2609.04	2542.98	2521.05	2388.63	−315.88	−11.68

**Table 3:** Estimation of ecosystem service function values (ESV) in the Dongting Lake area from 1995 to 2020.

Ecosystem Service Functions		ESV/100 Million						Change of ESV/100 Million	Growth Rate (%)
Primary Class Type	Secondary Class Type	1995	2000	2005	2010	2015	2020		
Supply service	Food production	98.58	96.78	95.05	92.76	91.74	89.97	−8.62	−8.74
	Raw material production	51.26	50.32	49.44	48.28	47.64	46.74	−4.52	−8.82
	Water supply	−24.04	−23.65	−23.09	−22.84	−21.94	−28.17	−4.13	17.18
Adjustment service	Gas regulation	195.01	191.45	188.08	183.65	181.26	178.01	−17.00	−8.72
	Climate regulation	434.44	426.50	419.09	409.26	403.66	395.57	−38.86	−8.95
	Environmental purification	172.16	168.99	166.09	161.99	160.30	153.65	−18.51	−10.75
	Hydrological regulation	1312.98	1288.46	1266.66	1232.79	1226.91	1132.20	−180.78	−13.77
Support service	Soil conservation	181.04	177.73	174.64	170.54	168.22	164.88	−16.16	−8.92
	Nutrient cycle	22.82	22.40	22.00	21.48	21.21	20.86	−1.96	−8.59
	Biodiversity	176.07	172.84	169.85	165.80	163.71	159.23	−16.84	−9.56
Cultural service	Esthetic landscape	84.20	82.65	81.23	79.26	78.34	75.70	−8.50	−10.10
Total value		2704.51	2654.47	2609.03	2542.97	2521.05	2388.63	−315.88	−11.68

## Data Availability

Not applicable.
